# Primary Raynaud's phenomenon in an infant: a case report and review of literature

**DOI:** 10.1186/1546-0096-9-16

**Published:** 2011-07-18

**Authors:** Anjali A Sharathkumar, Paul Castillo-Caro

**Affiliations:** 1 Department of Pediatrics, Children's Memorial Hospital, Northwestern University's Feinberg School of Medicine, Chicago, IL, USA; 2 Department of Pediatrics, Riley Hospital for Children, Indiana University School of Medicine, Indianapolis, IN, USA

## Abstract

Raynaud's phenomenon (RP) is an extremely unusual finding in early infancy. In the present report we describe a one-month-old previously healthy male infant who presented with unilateral acrocyanosis. Although infantile acrocyanosis is known to be a benign and self-resolving condition, it is generally bilateral and symmetric. The unilateral nature of the acrocyanosis was an atypical finding in this infant. Consequently, he was closely monitored to evaluate the progression of his acrocyanosis. Based on his benign clinical course and failure to demonstrate other etiologies contributing to his acrocyanosis, he was diagnosed to have primary RP. Due to the rarity of RP in children, we review the progress in understanding the pathophysiology, epidemiology and management of RP and additionally discuss the differential diagnosis of unilateral and bilateral acrocyanosis in infants.

## Background

Raynaud's phenomenon (RP) was first described by Maurice Raynaud in 1862 [1]. Classically, the initial description of RP involved triphasic color changes in the digits, with blanching (white) leading to cyanosis (blue) followed by reactive hyperemia (red) [2,3]. However, it has been realized that not every patient experiences all 3 phases of color change and the majority of patients present with uniphasic color change involving an isolated bluish discoloration of digits commonly known as acrocyanosis [4-6]. Unlike RP, acrocyanosis is a common phenomenon in infants and young children [4-7]. Acrocyanosis is generally bilateral, symmetric and involves hands and feet. Since infantile acrocyanosis is a benign and self-resolving condition, it does not require medical attention [7,8]. Rarely, acrocyanosis in infants can be caused by RP and may require immediate medical attention to prevent complications of RP [9-13]. In this report, we describe an infant who initially presented with unilateral acrocyanosis and was diagnosed to have primary RP based on his subsequent clinical course. In view of the rarity of RP in infants and young children, the literature about RP is reviewed with a specific focus on the pediatric population. To help differentiate benign acrocyanosis from acrocyanosis associated with other serious conditions, the differential diagnosis of unilateral and bilateral acrocyanosis in infants is also discussed.

## Case report

A one-month-old healthy male infant was brought to his pediatrician's office for the evaluation of bluish to blackish discoloration of his left hand. His mother incidentally noted this color change while she was changing his diaper. She did not recall any trauma or insect bite. She denied using naphthalene balls in the storage area for the infant's clothes. This history was helpful to exclude methemoglobinemia as exposure to naphthalene balls can cause infantile acrocyanosis [7]). He was breast-fed and his mother was the primary care taker. The infant's birth history was unremarkable except for neonatal physiological jaundice treated with phototherapy for 5 days. His past history was noteworthy only for a history of constipation. His family history was significant for ischemic heart disease at a young age (< 55 years old) in multiple members of his father's family. His father died at the age of 42 years due to a massive myocardial infarction. His mother had a history of migraines. He had three healthy siblings (three brothers, ages 11, 10 and 8 years).

Upon arrival at the outside hospital, he was an alert and healthy infant in no acute distress. His physical examination was normal including vital signs, growth and development except for acrocyanosis of his left hand with a clear demarcation at the wrist. His left palm was cooler than the rest of the extremities with sluggish capillary refill (~3 seconds). The peripheral and central pulses were equal and regular bilaterally. He was able to move all the extremities without any pain. The elevated arm stress test was negative for worsening of cyanosis or weakening of the radial pulse, thereby lessening the possibility of thoracic outlet syndrome. He was referred to a tertiary pediatric facility where he was admitted for further evaluation.

### Evaluation during hospitalization

Upper extremity duplex ultrasound, MRI/MRA/MRV of head, neck and left upper extremity were performed to rule out anatomical disturbances in vascular supply. Each test was negative. These results excluded several conditions, including thromboembolism, thoracic outlet syndrome contributing to compression of subclavian vein, vascular anomalies, and the presence of a mass or tumors in the region of cervical plexus including the stellate ganglion. A transthoracic echocardiogram confirmed normal cardiac anatomy and did not demonstrate any intracardiac mass, thrombus, or vegetation to suggest an embolic source for a presumed thrombotic event.

He underwent blood tests to detect infection and other systemic causes of acrocyanosis such as methemoglobinemia, polycythemia, antiphospholipid antibodies, and other hypercoagulable conditions. The complete blood count and comprehensive metabolic panel were normal. The erythrocyte sedimentation rate (ESR) and C-reactive protein (CRP) tests were also normal. His newborn screen for inborn errors of metabolism and hemoglobinopathies was negative. His coagulation tests (PT and aPTT) were normal and antiphospholipid antibodies and antinuclear antibody (ANA) assays were negative. He also underwent testing for inherited thrombophilia such as factor V Leiden mutation, prothrombin gene mutation, and methylene tetrahydrofolate reductase (MTHFR) mutation. Results were positive for homozygosity for a MTHFR C677T mutation with normal homocysteine levels.

Due to the concerns about the transplacental transfer of maternal antibodies contributing to development of RP in this young infant, it was decided to evaluate his mother for other medical conditions associated with RP [14-16]. Her blood work up revealed no evidence for systemic lupus erythematosus (antiphospholipid antibodies, ANA, antidsDNA), scleroderma (antitopoisomerase 1 antibodies) or cryoglobulinemia (cryoglobulin levels).

### Course during hospitalization and outpatient follow up

At admission, he was begun on low molecular weight heparin (LMWH, dose: 1 mg/kg/dose, q 12 hourly) due to the concerns of a thromboembolic event. He showed gradual improvement in discoloration of his hands over next 72-hours. Since there was no evidence of thromboembolism, his LMWH treatment was discontinued after three days and it was decided to follow him closely in an outpatient clinic without any further medical intervention. Over the next few months he continued to have 1-2 self-resolving episodes of acrocyanosis per month, involving both hands along with bluishness of tip of the nose, lips, ears and periumbilical area and each lasting for a few minutes to an hour. They were not associated with cold exposure but were felt to be precipitated by abdominal colic due to constipation. At 9 months of age his initial tests for SLE, and other rheumatic diseases was repeated. This evaluation was normal.

Based on his clinical course, he was diagnosed to have "primary Raynaud's phenomenon". His treatment included laxatives for constipation and close clinical monitoring. Over the next one year, he continued to have occasional episodes of acrocyanosis without any medical consequences. At his last follow up (age 2 years), his growth and development was normal (length and weight at 90th percentile).

## Discussion

The infant described in this report presented with a unilateral acrocyanosis at one month of age. He was diagnosed to have primary RP at 9 months. This diagnosis was based on his clinical course and exclusion of other causes of unilateral acrocyanosis including vascular anomalies, thromboembolism and thoracic outlet syndrome. (Table 1) [7]. Even though acrocyanosis is very common condition in newborn period, involvement of only one hand was an atypical finding for infantile acrocyanosis. This finding provided a clue to consider the possibility of RP in the differential diagnosis.

**Table 1:** Causes of acrocyanosis

Type of acrocyanosis	Causes
Unilateral acrocyanosis	
	Local trauma/digital injury
	Local infection: Paronechia
	Raynaud's phenomenon
	Thoracic outlet syndrome
	Carpel Tunnel syndrome
	Cervical tumors compressing neurovascular bundle* e.g. Neuroblastoma, stellate ganglion tumor
	Thromboembolism of arteries of palmar arch
	Frostbite
	Reflex Sympathetic dystrophy
Bilateral acrocyanosis**	
	Benign acrocyanosis of infancy
	Frostbite
	Pernio
	Raynaud's phenomenon
	Reflex sympathetic dystrophy
	Hypoxemia**
	Congenital cyanotic heart disease**
	Methemoglobinemia**
	Purpura fulminans
	Pheochromocytoma

*: Need to look for other signs of Horner's syndrome on the ipsilateral side of the face (ptosis, anhydrosis, miosis of pupil or unequal pupils, poor light reflex and enopthalmos);**: Need close clinical evaluation for presence of central cyanosis.

Over the last decade significant advances have been made in understanding the pathophysiology of RP [17-21]. Irrespective of the underlying etiology, RP is manifested via vasospasm of the small muscular arteries and arterioles of the digits [18]. Similar to benign acrocyanosis of infancy, RP is also triggered by exposure to cold and emotional stress. It can be asymmetric and may last longer than benign acrocyanosis. Based on the available data, an over- activity of the sympathetic nervous system along with an imbalance of vasodilator and vasocontrictor substances may be the most likely etiology for RP [18]. In patients with RP, digital cutaneous neurons show a deficient release of a potent vasodilator, the calcitonin-gene related peptide. This primary pathology may be exaggerated by other factors as well, some of which are influenced by cold or emotional triggers. For example, in response to cold, various vasoconstricting substances such as catecholamines, endothelin-1, and 5-hydroxytryptamine are released. These chemical mediators could cause digital artery vasoconstriction and the symptoms of RP. In some cases, this could trigger a cascade of neutrophil and platelet activation, which through the release of inflammatory agents such as endothelin-1 and TNF-alpha, contribute to the endothelial damage seen with more severe RP [21]. There is some suggestion that elevated levels of homocysteine, a sulfur amino acid that is proposed as an independent risk factor for atherosclerosis, may have an association with RP [17,22,23]. RP appears to have a strong familial component suggesting a genetic link, though this link is yet to be clarified [20]. It is also unclear if constipation can exaggerate the imbalance of vasodilator and vascontrictor substances.

Raynaud's phenomenon is traditionally classified as 'primary' (previously known as Raynaud's disease) or 'secondary' [24]. Primary RP is diagnosed when it occurs in the absence of associated diseases. In contrast, secondary RP is diagnosed in the presence of a well-defined disease states such as SLE, polyarteritis nodosa (PAN) or scleroderma (Table 2). [25-28] Primary RP is generally a benign condition [13], but secondary RP can lead to significant morbidity, including digital gangrene [29] and may be life-threatening [9]. Of those patients with primary RP, ~13% of patients will eventually be diagnosed to have secondary RP [11]. Although it is difficult to predict which patients will be eventually diagnosed to have secondary RP, children with secondary RP can show changes in their nailfold capillaries. The direct observation of the nailfold microvasculature with videocapillaroscopy is useful for suspecting secondary RP earlier during the clinical course [30,31]. Generally, the presence of giant capillaries, avascular fields and irregular architecture of nailfold capillaries is predictive of the development of SLE, PAN, or scleroderma in patients with RP [32]. According to Allen and Brown's criteria of minimum diagnosis, a negative antinuclear antibody titer and negative findings on capillaroscopy are the most reliable way to distinguish between primary and secondary RP [33]. Since our patient's clinical presentation (age, sex, clinical features) and blood workup was consistent with primary RP, he was not evaluated for capillary nailfold abnormalities.

**Table 2:** Causes of Raynaud's phenomenon

Category	Disease conditions
Connective tissue/autoimmune disorders	Scleroderma, Dematomyositis, Systemic lupus erythematosus, Rheumatoid arthritis, Sjogren's syndrome Antiphospholipid antibodies (APLA) syndrome Polyarteritis nodosa
Repetitive trauma	Work involving tools with vibrations
Drugs	Interferon-alpha, Bleomycin Ergotamine
Infections	Helicobacter Pylori Parvovirus B19
Metabolic	Atherosclerosis Cryoglobulinemia Hyperhomocysteinemia
Chemical exposure	Vinyl chloride exposure in plastic industry Smoking
Other	Carpal-tunnel syndrome CREST* syndrome

* CREST: calcinosis, Raynaud's phenomenon, esophageal dysmotility, sclerodactyly, and telangiectasia.

Since RP is extremely rare in children, specifically in infants, the knowledge about its epidemiology, clinical spectrum and the natural evolution is quite limited [9,11-13,29,34]. The first description of RP in children appeared in 1967, almost 100 years after the initial description of RP by Raynaud in 1862 [1]. This report described a series of 6 children (ages, 2.5 to 5 years) with classic RP [13]. Since 1967, there are only a handful of reports of RP in children [9,11,29,34]. In general, female children are more predisposed to development of RP and the onset of RP generally occurs around menarche implying the influence of ovarian hormones in the pathogenesis of this entity [4,11,34]. Primary RP is more common in children than secondary RP [11,34]. Earlier reports in children suggest the association of RP with rheumatic diseases in children [34,36]. Similar to adult literature, pediatric studies suggest that positive ANA and abnormalities of nailfold capillaries may be associated with secondary RP [34].

The largest cohort study in children provided more insight into the epidemiology of RP in children and showed that RP is highly heterogeneous in children [11]. Although exposure to cold was the primary trigger in the majority, (~70% of children) ~10% did not have any known trigger. Primary RP followed a bimodal pattern of age of onset affecting young infants and teenage population. Half of these children experienced additional symptoms such as pain, tingling, and numbness. Interestingly, 11% (9/82) of children with primary RP were lmisdiagnosed as "acrocyanosis" and 4 of them were younger than 2 years of age. These 4 children uniformly experienced monophasic or biphasic color change involving the whole hand, foot, or both under the influence of cold or without an apparent cause. There are only two case reports describing the RP in infants [9,29]. Both these infants presented at 5 months of age with severe disease and required treatment with vasodilators. A patient reported by Sayre initially presented with predominant involvement of right foot [29]. Her symptoms were uniphasic and lasted for 72 hours prior to presentation. At the age of 9 months, she showed involvement of fingers as well. An infant described by Krigel et al. [9], presented with acrocyanosis of toes for 3 days prior to admission and showed classic RP with triphasic color changes. The infant progressed to the development of digital gangrene. This patient died at the age of 8 months due to vasomotor collapse. Her autopsy studies revealed the diagnosis of PAN thus supporting secondary RP as the underlying cause of her acrocyanosis. Our patient presented at an earlier age with mild symptoms but his cyanosis lasted for almost 48-72 hours, similar to both these cases. As transient APLAs have been described in pregnant women [37], our initial work up was focused on evaluating him and his mother for these antibodies to rule out transplacental transfer of these antibodies contributing to the clinical presentation.

Our patient was evaluated for genetic risk factors of thrombosis due to the concerns about unilateral thrombosis at his presentation and the family history of young age heart attacks [38]. He was homozygous for MTHFR C677T mutation. MTHFR mutation can be associated with hyperhomocysteinemia [39] and high homocysteine levels are shown to be associated with decreased vasodilation both in animal models [40] and in humans [41]. On the same note, patients with RP are shown to have elevated homocysteine levels compared to normal controls [42]. In our case the patient's homocyteine levels were normal, making this a less likely etiology for his RP. However, whether MTHFR mutation in itself plays a direct role in vascular instability is yet to be clarified.

Our patient was also evaluated for systemic causes of central cyanosis such as methemoglobinemia and congenital cyanotic heart disease. Generally this evaluation is not required for children with unilateral acrocyanosis. However, maternal anxiety and inability to provide a long-term prognosis of this infant forced the medical team to perform the extensive evaluation. Although during two-years of follow up, there was no indication of the other disorders which might be causal for the development of RP, it is possible that in future his underlying condition may become clinically evident.

Management of RP is generally supportive and relies upon its accurate diagnosis. The mild forms of primary RP can be controlled by non-pharmacologic approaches such as avoidance of exposure to cold or emotional stress. In moderate to severe cases, vasodilator therapy including calcium channel blockers, either systemic or topical, is required to relieve the vasospasm. Rarely prostacycline infusions [43], antiplatelet agents [44,45] and antithrombotic therapies [46] have been used with variable success.

Surgery is reserved for extreme cases and generally involves digital sympathectomy [47]. In the severe forms of the disorder, intravenous infusion of prostacyclin as well as endothelin-1 receptor antagonists and specific inhibitors of phosphodiesterase-5 are emerging as the treatment of choice [48,49]. Investigational agents for the treatment of RP include selective alpha-2c adrenergic receptor blockers, inhibitors of protein tyrosine kinase and Rho-kinase, as well as calcitonin gene-related peptide. In patients with secondary RP, treatment of underlying disease is critical to control the episodes of RP.

## Conclusion

This case report highlights that despite an improved understanding of the pathophysiology of RP, the diagnosis of primary RP in infants is challenging and It can take weeks to months to confirm the diagnosis. The rarity of its occurrence in infants, the similarities in clinical presentation between primary RP and benign acrocyanosis of infancy, and the absence of diagnostic tests confirming the diagnosis of primary RP contribute to delay in the diagnosing of RP. Clinical clues that should alert the clinician to suspect RP in infancy include the presence of atypical features such as a prolonged acrocyanosis episode (> 72 hours) and/or a unilateral acrocyanosis. A high index of suspicion and close clinical monitoring is required to ensure an accurate diagnosis and appropriate clinical management.

## Consent

Written informed consent was obtained from the patient for publication of this case report. A copy of the written consent is available for review by the Editor-in-Chief of this journal.

## List of abbreviations

ANA: Antinuclear antibodies; ANCA: Antineutrophil cytoplasmic antibodies; APLA: Antiphospholipid antibodies; CRP: C-reactive proteins; ESR: Erythrocyte sedimentation rate; LMWH: Low Molecular Weight Heparin; MTHFR: Methylene Tetrahydrofolate reductase; MRI: Magnetic Resonance Imaging; MRA: Magnetic Resonance Angiogram; MRV: Magnetic Resonance Venography; ANCA: Antineutrophil cytoplasmic antibodies; PAN: Polyarteritis nodosa; PT: Prothrombin time; aPTT: Activated Partial thromboplastin time; RP: Raynaud's phenomenon; TNF: Tumor necrosis factor.

## Conflict of interest

The authors declare that they have no competing interests.

## Contributions of Authors

PC and AS conceived the idea and wrote the manuscript. Both authors read and approved the final manuscript.
